# Changes in *Mycobacterium tuberculosis*-Specific Immunity With Influenza co-infection at Time of TB Diagnosis

**DOI:** 10.3389/fimmu.2018.03093

**Published:** 2019-01-04

**Authors:** Joseph Mendy, Sheikh Jarju, Rhiannon Heslop, Adama L. Bojang, Beate Kampmann, Jayne S. Sutherland

**Affiliations:** ^1^Vaccines & Immunity Theme, MRC Unit The Gambia at the London School of Hygiene & Tropical Medicine, Banjul, Gambia; ^2^Faculty of Biosciences, The University of Manchester, Manchester, United Kingdom

**Keywords:** tuberculosis, influenza, bacterial load, cytokines, flow cytometry

## Abstract

**Background:** Prior Influenza A viral (IAV) infection has been shown to increase susceptibility to tuberculosis (TB) and TB has also been shown to be a primary cause of death during pandemics, including the Spanish Influenza outbreak of 1918–1919. The majority of data has been obtained from mouse models, thus the aim of this study was to determine the impact of Flu co-infection on host immunity and disease severity in TB patients at diagnosis.

**Methods:** Sputum from 282 patients with active TB were analyzed for presence of FluA/FluB RNA at presentation using multiplex PCR. Sputum RNA was also analyzed for *Mycobacterium tuberculosis* (Mtb) load using ^16^S RNA amplification. Supernatants from digested sputum and Mtb antigen-stimulated whole blood were analyzed using multiplex cytokine arrays and PBMC were analyzed for cytokine production from CD4+ T, CD8+ T and Mucosal Associated Invariant T cells (MAITs).

**Results:** 12 (4.3%) of TB patients were found to have FluA or FluB viral RNA present in their sputum at the time of TB diagnosis. The TB/Flu co-infected patients had a significantly higher bacterial load compared to those with TB mono-infection (*p* = 0.0026). They had lower levels of IL17A in *ex vivo* sputum (*p* = 0.0275) and higher MCP-1 (CCL2) levels in the blood following PPD stimulation (*p* = 0.0267). TB/Flu co-infected subjects had significantly higher IFN-γ+IL-17+CD4+ and IFN-γ+IL-17-CD8+ cells compared to TB mono-infected subjects.

**Conclusions:** These data show that Flu co-infection at time of TB diagnosis is associated with a higher bacterial load and differential cellular and soluble profiles. These findings show for the first time the impact of TB/Flu co-infection in a human cohort and support the potential benefit of Flu vaccination in TB-endemic settings.

## Introduction

Tuberculosis (TB) and influenza are two of the greatest threats to global health (1) Recent data has indicated a role for TB in characterizing the age-specific risk of death during the 1918–1919 Spanish influenza (H1N1) outbreak (2) while in the 2009 H1N1 pandemic, 10% of patients who died in a South African cohort also had active TB (3) Interestingly, while prior infection with influenza undoubtedly increases susceptibility to secondary bacterial infections, it has also been suggested that a reduction in immunity associated with Mtb infection increases susceptibility to Influenza (4) A more recent study from South Africa indicated that co-infection with influenza is associated with an increased risk of death compared to those hospitalized with TB mono-infection (5) However, these observational studies have not provided mechanistic insights.

Mtb is inhaled through air droplets to reside within macrophages and neutrophils in the lung (6) Active TB disease is characterized by a Type I IFN response, which modulates the protective effects of IFN-γ by increasing production of IL-10 and reducing downstream mediators induced by vitamin D (7) Alveolar macrophages are also the prime target for Influenza virus infection (8), causing their depletion and thus loss of the first line of defense against Mtb infection. Increased production of Type I interferons (IFNα/β) as part of the anti-viral response inhibits the downstream effects of Type II interferon (IFN-γ) responses known to be critical for Mtb control (9) IFNα/β-mediated signaling can promote the production of high levels of IL-10, leading to reduction in macrophage function (10), as well as the induction of pro-apoptotic factors such as TRAIL resulting in loss of epithelial cells (11) Experimental animal models have shown that influenza infection significantly enhances susceptibility to secondary infection with bacterial species, culminating in increased bacterial loads and reduced survival in co-infected animals (12) Recent data has shown that Influenza vaccination can reduce the incidence of TB (13) and this may be mediated through Th17 cells (14).

Despite these findings, there is a lack of understanding of the underlying immunological mechanisms of TB/Flu co-infections in humans. Thus, this study aimed to determine how TB/Flu co-infection influences immunity to Mtb antigens by determining the impact of Flu co-infection on mycobacterial burden, lung inflammatory profiles and Mtb-specific immunity in TB patients using multiparameter flow cytometry and luminex profiling.

## Methods

### Ethics Statement

This study was carried out in accordance with the recommendations of MRC/Gambian Government joint ethics committee with written informed consent from all subjects where blood samples were taken. Samples for routine diagnostic evaluation, including multi-pathogens, do not require written informed consent. The protocol was approved by the MRC/Gambian Government joint ethics committee (SCC 1333).

### Subjects and Samples

Two hundred and eighty-two sputum samples used in this study were obtained during routine diagnostic evaluation and confirmed to be MTB complex by smear and/or liquid sputum culture. An aliquot of sputum was digested with an equal volume of Sputolysin (MerckMillipore, USA) for 15 min and centrifuged. The supernatant was harvested and the pellet resuspended in Trizol (ThermoFisher, UK) and stored at −80°C until use. For subjects subsequently recruited to a research study, a heparinised blood sample was also collected following written informed consent.

### Whole Blood Antigen Stimulation

Four hundred and fifty microliter whole blood was incubated with 50 μl of: phosphate buffered saline (PBS) as negative control (NIL);or with 50 μl of phytohaemagglutinin (PHA; 5 μg/ml) as positive control; purified protein derivative (PPD; final concentration 10 μg/ml; Staten Serum Institute, Denmark) or early secretory antigenic target 6, culture filtrate protein 10 fusion protein (ESAT-6/CFP10; EC; final concentration 10 μg/ml; kindly provided by Prof. THM Ottenhoff, Leiden University Medical Center (LUMC), The Netherlands). Following overnight incubation at 37°C, 5% CO_2_, supernatant was collected and stored at −80°C prior to use.

### Multiplex Cytokine Arrays

Multiplex immunoassays were carried out using the 27-plex Bio-Plex Pro™ Human Th1/Th2 Cytokine Panel (Bio-Rad, Belgium) according to the manufacturer's instructions. Analytes measured were IL-1β, IL-1ra, IL-2, IL-4, IL-5, IL-6, IL-7, IL-8, IL-9, IL-10, IL12p70, IL-13, IL-15, IL-17A, eotaxin, basic FGF, G-CSF, GM-CSF, IFN-γ, IP-1O, MCP-1, MIP-1α, MIP-1β, PDGF-BB, RANTES, TNF-α, and VEGF. Briefly, lyophilized standards were reconstituted and serial dilutions performed. Coupled beads were diluted in assay buffer and 50 μl added to each well of the assay plate. Fifty microliter of diluted standards, blanks, samples (stimulated whole blood supernatants or *ex vivo* sputum) and controls were added per well. Plates were then incubated at room temperature (RT), with shaking at 350 rpm, for 30 min followed by 3 washes in wash buffer. Detection antibodies were diluted to 1 in 20 of their original concentration in detection antibody diluent and 25 μl added to each well followed by another 30 min incubation. Following 3 washes, streptavidin-PE was diluted to 1 in 100 in assay buffer and 50 μl added to each well. Plates were then incubated for 10 min and washed 3 times. One hundred and twenty-five microliter assay buffer was then added to each well, plates were briefly shaken and subsequently read using Magpix plate reader, with Bio-Plex Manager Software (version 6.1; Bio-Rad, Belgium). No significant differences were observed in background levels within or between groups. Thus, all cytokine responses for NIL stimulated samples were subtracted from those for blood incubated with EC and PPD antigens.

### Molecular Bacterial Load Assay

#### Preparation of Mtb Standards

Five hundred microliters of wild-type Mtb (H37Rv) stock and 800μl of mycobacteria growth indicator tube (MGIT) growth supplement were added to a MGIT tube and incubated in a BACTEC MGIT 960 (Becton Dickenson, USA) machine for 5 days. Viability was confirmed via a fluorescent reaction in the MGIT tube. The tube was mixed by hand, and 500 μl was inoculated into 20 μl 7H9 with TWEEN and incubated at 37°C. Optical density (OD) was measured using a spectrophotometer every 2 days to estimate the growth of the bacteria in conjunction with the McFarlane scale. Once an OD of 2.2 was reached 1 ml aliquots of the suspension were frozen at −80°C in Trizol. To confirm the top standard concentration, 10-fold serial dilutions of 10^−1^ to 10^−5^ were performed with 7H9 media on one aliquot. Three 20 μl drops were plated onto 7H11 agar and incubated at 37°C for 3 weeks and colony forming units (CFU) were counted.

#### Extraction of RNA

Before extraction, 2 μl of 560 RNA Internal Control RNA (Bioline, UK) was spiked into 1 ml sputum samples in Trizol. Two hundred microliters of chloroform was then added to each tube, samples were mixed vigorously and incubated at room temperature (RT) for 10 min. Samples were then centrifuged at 13,000 rpm for 15 min and the upper aqueous phase was transferred to fresh tubes. An equal volume of 70% ethanol (approximately 600 μl) was added to each tube and mixed vigorously. The sample solutions were then transferred to RNeasy MiniElute Spin Columns (Qiagen, Netherlands) and RNA purified according to Qiagen protocol. For the standards, RNA was extracted and 10-fold serial dilutions performed using nuclease free water (Qiagen, The Netherlands).

#### Quantitative PCR

Levels of ^16^S RNA and internal control (IC) were quantified using reverse transcription polymerase chain reaction (RT-PCR). To detect ^16^S RNA, a master mix containing 12.5 μl Quantitect Master Mix, 6.65 μl of nuclease free water, 0.25 μl reverse transcriptase, 0.3 μl of ^16^S-ROX (Rox-AGGACCACGGGATGCATGTCTTGT-BHQ2) (all supplied by Qiagen, Netherlands) per reaction was prepared. A master mix containing 12.5 μl Quantitect Master Mix, 5.05 μl of nuclease free water, 0.25 μl reverse transcriptase, 1.2 μl 50 nM MgCl^2+^ (Qiagen, The Netherlands) and 1 μl VIC labeled 560 Control Mix (Bioline, UK) per reaction was prepared to detect the IC. Five microliter of RNA standards, samples or H_2_0 were added to the 96 well plate in triplicates; 20 μl of ^16^S-ROX mastermix was added to two wells, 20 μl of IC mastermix was added to the third. The plate was briefly vortexed, then centrifuged at 10,000 rpm for 30s before being placed in 7500 Real-Time PCR System (Applied Biosystems, USA). The cycling parameters were set to 50°C for 30 min, 95°C for 10 min, then 45 cycles of 95°C for 15 s and 60°C for 1 min. Analysis was performed on ABI 7500 software (version 2.3, Affymetrix, USA).

### Respiratory Pathogen Multiplex qPCR

rRT-PCR was performed using a Fast Track Diagnostic (FTD) kit (Junglister, Germany) for Influenza A (FluA) and Influenza B (FluB). The reaction mix consisted of a primer-probe mix for FluA/B, Fast-track 2X enzyme mix and buffer. A 25 μl total reaction volume consisted of 10 μl RNA template or controls (plasmid pool control and a negative RT control with water only) and 15 μl reaction mix. The detector wavelengths used were 520 nm for FluA and 610 nm for FluB. The rRT-PCR one step conditions were 42°C for 15 min, 94°C for 3 min and 40 cycles of 94°C for 8 s and 60°C for 34 s. PCR was performed using ABI QuantStudio 7-flex real-time PCR system (Thermo Fisher, USA).

### Multiparameter Flow Cytometry

Cryopreserved PBMC samples were thawed and resuspended in RPMI+10%FCS+0.02% benzonase. After 6 h rest, cells were counted and resuspended at 0.5–1.0 × 10^6^ cells per test in 500 μl in polystyrene tubes and incubated at 37°C, 5% CO_2_ for 16–20 h with 1X cell stimulation cocktail plus protein transport inhibitors (CSC; eBioscience, UK), containing phorbol 12-myristate 13-acetate (PMA), ionomycin, brefeldin A and monensin. Negative controls were incubated in the same conditions without the PMA but with a corresponding 1X protein transport inhibitor cocktail (PTI; eBioscience, UK). After overnight stimulation, tubes were centrifuged at 1500 rpm for 5 min and the supernatant removed. Cells were incubated with Live/Dead Aqua (eBioscience, UK) for 10 min at RT, in the dark. Cells were washed with 1 ml FACS buffer (PBS, 1% FCS, 0.2% Na Azide, 0.1% EDTA) and centrifuged at 1500 rpm for 5 min. Supernatant was removed, a cell surface cocktail of anti-human CD3 allophycocyanin-cyanine 7 (APC-Cy7), CD8 Alexa Fluor 700 (AF700), CD161 phycoerythrin (PE), Vα7.2 allophycocyanin (APC) (all from eBioscience, UK) and CD26 fluorescein isothiocyanate (FITC) was added and incubated for 15 min at 4°C. Cells were then washed in 1 ml FACS buffer and centrifuged at 1500 rpm for 5 min, 150 μl of Cytofix/Cytoperm solution was added (Becton Dickinson, USA) and incubated for 15 min at 4°C. Another wash step was performed and cells were incubated with 1X Perm/Wash buffer (BD, USA) for 20 min at RT, in the dark. Cells were washed and centrifuged at 1800 rpm for 5 min, supernatant removed and intracellular cytokine staining performed with IFN-γ phycoerythrin-CF594 (PE-CF594) and IL-17 phycoerythrin-cyanine 7 (PE-Cy7) made up in 1X Perm/Wash buffer (all from BD, USA). Cells were incubated for 30 min at RT, in the dark, washed once and resuspended in 300 μl FACS buffer. 200,000 lymphocytes were acquired per sample using a LSR III Fortessa flow cytometer (BD Biosciences, USA) and BD FACSDiva software. Resultant FACS plots were analyzed using FlowJo (Version 10.1; Treestar, USA).

### Statistics

A Mann-Whitney U test was used to compare bacterial load, cytokine profiles and flow cytometry cell subsets in TB/Flu and TB patients. All statistical analysis was carried out with GraphPad Prism v7.0 (Software MacKiev, USA).

## Results

### Participant Information

We analyzed sputum from 282 TB cases and found 12 (4.3%) were co-infected with FluA/B at the time of presentation to the TB clinic at MRCG. Of these, 75% were FluA and 25% FluB with 50% of subjects presenting in September (i.e., the rainy season). While the majority of samples were obtained for diagnostic/screening purposes with no corresponding demographic data available, 86 patients were subsequently enrolled in our research studies and thus had demographic information available. No differences in age or sex were seen between the groups with a median age of 31 [Interquartile range (IQR) 22–45] for co-infected subjects (*n* = 6) and 28 [21–38] for mono-infected subjects. There were more males than females in both groups, which is common in The Gambia for TB patients. Known HIV-positive subjects were excluded from the study. All patients were microbiologically cured by 6 months but were not followed longer than this to determine morbidity/mortality rates or relapse/re-infection rates.

Out of 37 non-TB (ie patients with other respiratory diseases) analyzed, only 1 was infected with influenza and were thus not included in downstream analyses. They were also significantly older than the TB mono or co-infected groups (76 years). We performed some pilot analysis of the ORD group and did not find any Flu associated with other infections including bacterial pneumonia, although we did see some co-infection of pneumonia with TB. However, numbers were too small (*n* = 20) for any conclusions to be drawn.

### TB/Flu Co-infection Is Associated With a Higher Bacterial Load at Diagnosis

**Figure 1:**
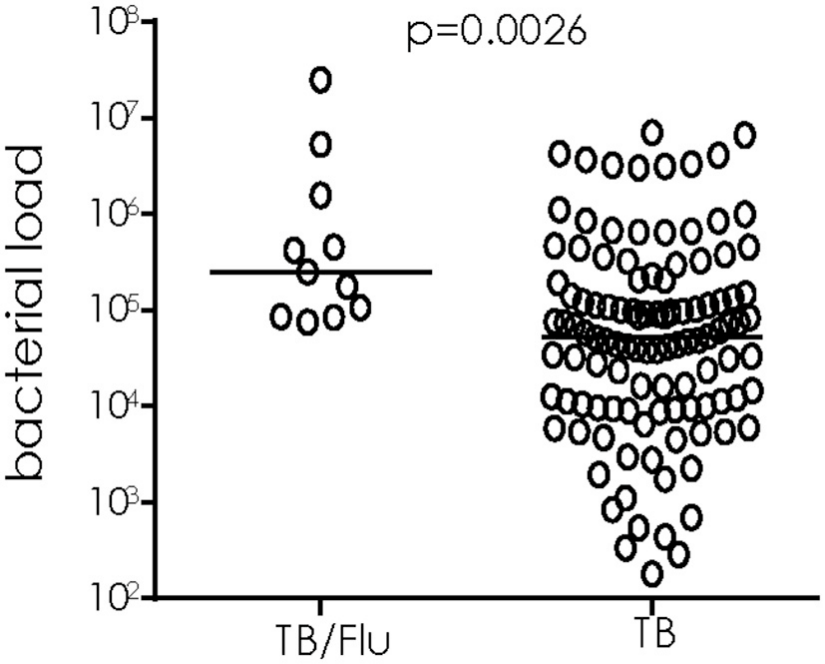
TB/Flu co-infection significantly increases bacterial load in sputum at diagnosis. ^16^S rRNA analysis was performed on sputum samples from TB/Flu co-infected (*n* = 11) and TB mono-infected (*n* = 110) patients at baseline. Bacterial load was calculated from a standard curve using H37Rv reference strain. Data were analyzed using Mann-Whitney *U*-test. Bar indicates median.

We used ^16^S rRNA mycobacterial load assay to determine viable bacilli counts in sputum samples of TB/Flu co-infected subjects as recently published by our laboratory (15) We saw a significant increase in bacterial load in TB/Flu co-infected patients compared to those with TB mono-infection (median [Interquartile range (IQR)] 2.5E5 [0.9E5-16E5] cfu/ml for co-infected compared to 0.5E5 [0.1E5-2E5]; *p* = 0.0026; Figure 1).

#### TB/Flu Co-infection Influences Cytokine Levels in *Ex vivo* sputum and Mtb-antigen Stimulated Blood

**Figure 2:**
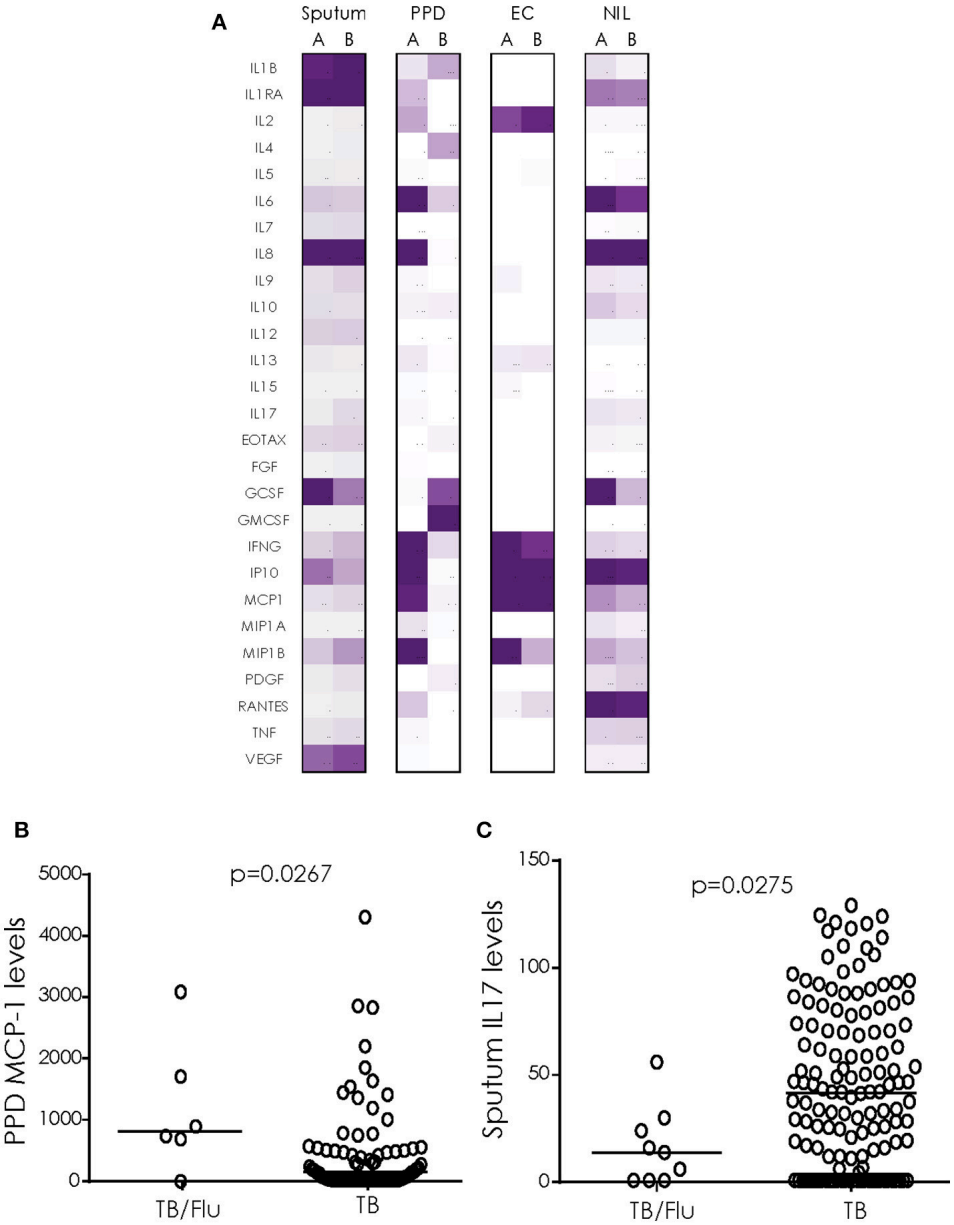
Cytokine profiles in TB/Flu Co-infection. **(A)** heat map showing differences in 27 cytokines/chemokines in sputum (*n* = 11 co-infected and *n* = 135 mono-infected) and antigen-stimulated blood (*n* = 6 co-infected and *n* = 80 mono-infected) from TB/Flu co-infected **(A)** and TB-mono-infected **(B)** patients. **(B)** MCP-1 levels in whole blood PPD-stimulated supernatants and **(C)** IL-17 levels in *ex vivo* sputum (both pg/ml). Data were analyzed using Mann-Whitney *U*-test; bar indicates median.

We next analyzed cytokine/chemokines in antigen-stimulated whole blood and *ex vivo* sputum from subjects who also had a paired blood sample available. In general, unstimulated (*ex vivo*) sputum had higher levels of most cytokines compared to blood stimulated overnight with EC or PPD (Figure 2A). EC and PPD stimulated blood showed higher levels of IFN-γ, IP-10 and MCP1 compared to sputum and NIL samples (Figure 2A). MCP-1 levels were significantly higher in TB/Flu co-infected compared to TB mono-infected subjects following PPD stimulation (*p* = 0.0267; Figure 2B). We also saw a significantly lower level of IL-17A in *ex vivo* sputum from TB/Flu co-infected compared to TB mono-infected patients (*p* = 0.0275; Figure 2C).

#### TB/Flu Co-infection Influences Immune Cell Subsets

**Figure 3:**
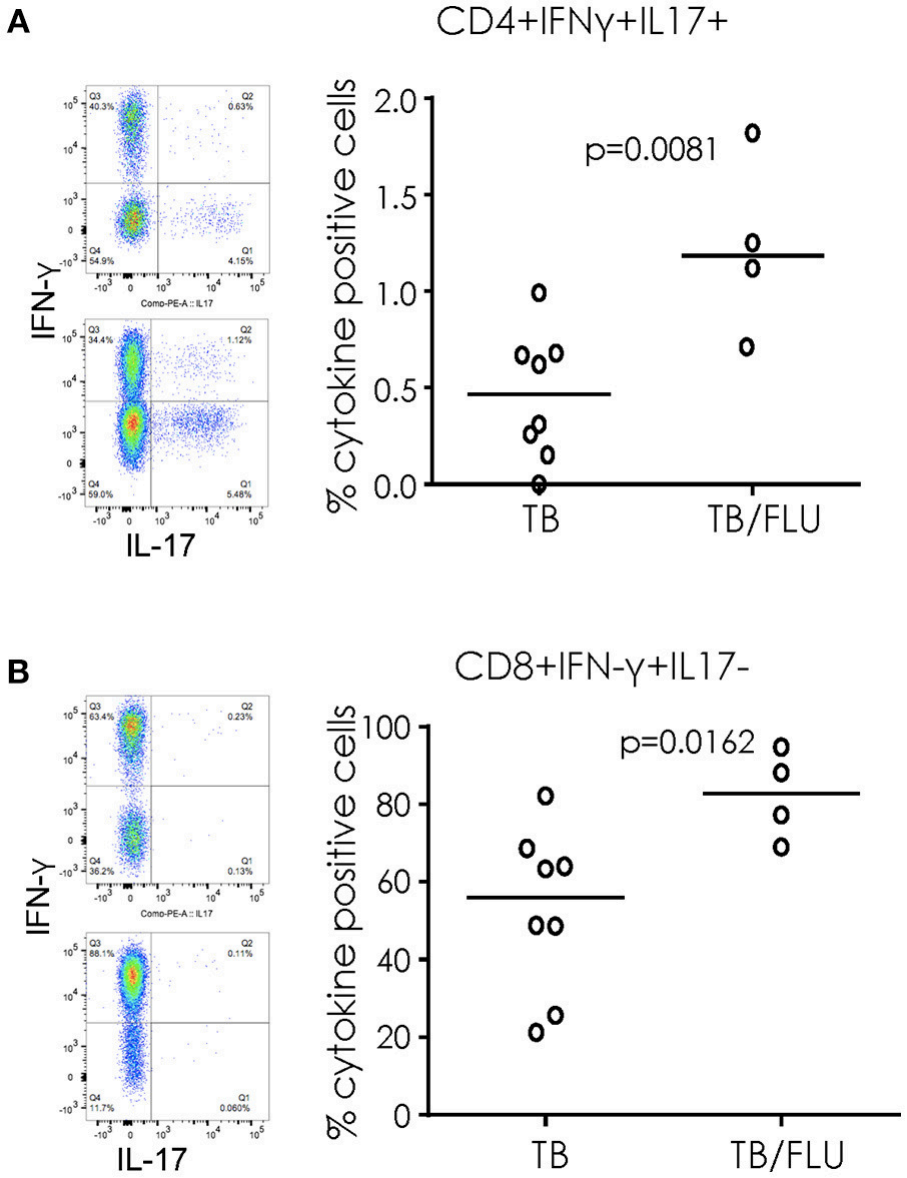
Increased cellular cytokines in TB/Flu co-infection. IFN-γ and IL-17 production from **(A)** CD4+ and **(B)** CD8+ T cells following PMA stimulation. On the left are representative flow cytometry plots showing IFN-γ (y-axis) and IL-17 (x-axis). Top is TB mono-infected and bottom is TB-Flu co-infection. The graphs on the right show grouped analysis. Data were analyzed using Mann-Whitney *U*-test. Bar indicates median.

**Figure 4:**
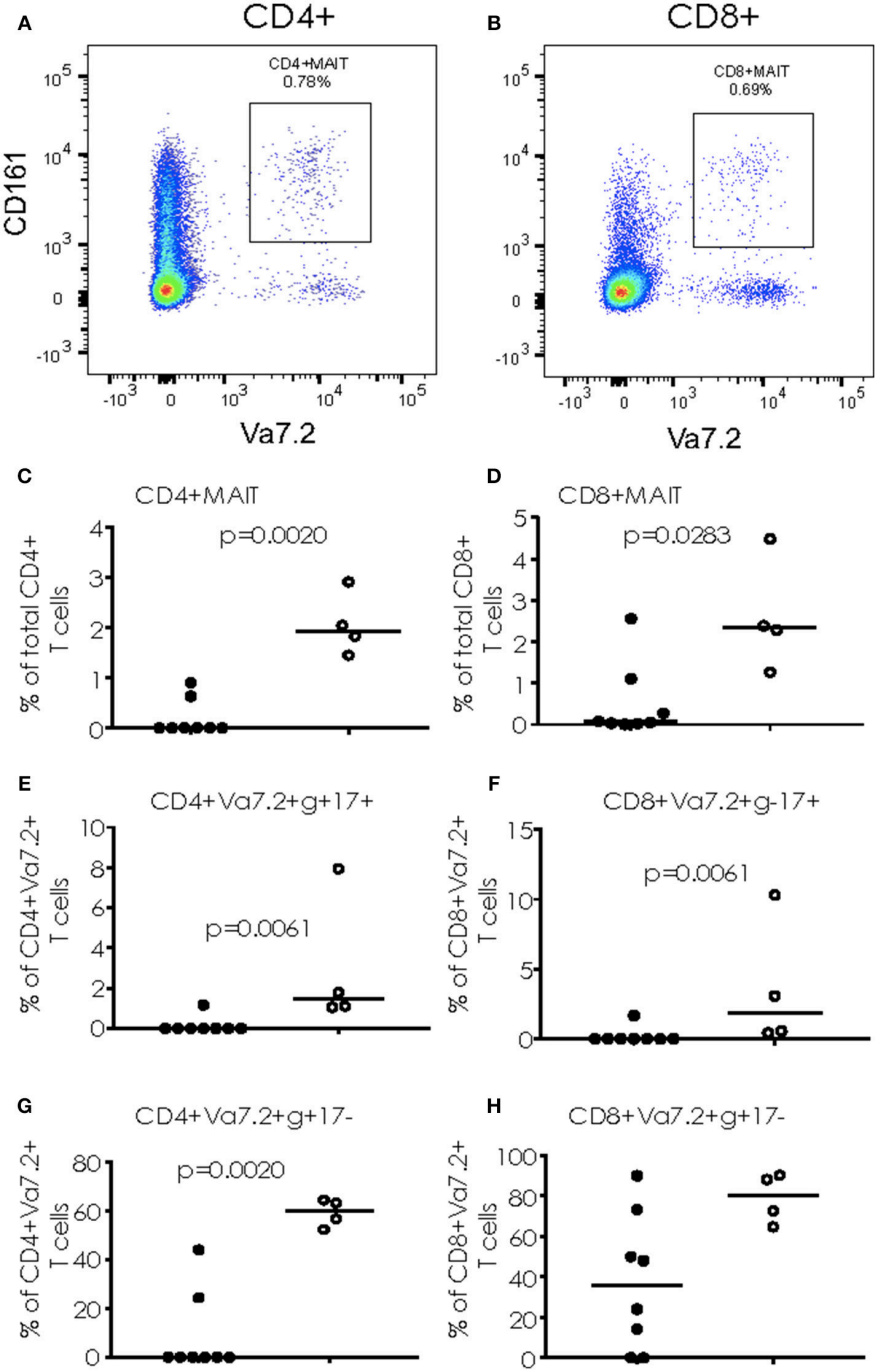
Cytokine production from MAIT cells. **(A,B)** Representative FACS plots showing CD4+CD161+Va7.2+ **(A)** and CD8+CD161+Va7.2+ **(B)** cells. **(C,D)** total CD4+ and CD8+ MAIT cells. (**E,F)** IL-17+ MAIT cells and **(G,H)** IFN-γ+ MAIT cells. Data were analyzed using Mann-Whitney *U*-test. Bar indicates median. Black dots = TB mono-infected subjects; white dots = TB/Flu co-infected subjects.

When cryopreserved PBMC were analyzed by flow cytometry, we saw a significant increase in the proportion of CD4+ T cells simultaneously producing IFN-γ and IL17 (*p* = 0.0081) and CD8+ T cells producing IFN-γ alone (*p* = 0.0162) following overnight stimulation with PMA/Ionomycin in TB/Flu compared to TB only patients (Figures 3A,B). In addition, TB/Flu co-infected subjects had a significantly higher proportion of CD4+Vα7.2+CD161+ and CD8+Vα7.2+CD161++ (MAIT) cells compared to TB mono-infected subjects (*p* = 0.0020 and *p* = 0.0283 respectively; Figures 4A–D). When functionality of each population was assessed, we saw significantly higher levels of CD4+Vα7.2+ cells producing both IFN-γ and IL-17 and those producing IFN-γ alone in TB-Flu co-infected subjects compared to TB mono-infected subjects (*p* = 0.0061 and *p* = 0.0020 respectively; Figures 4E,G). Similarly, TB/Flu co-infected subjects had significantly higher proportion of CD8+ MAIT cells producing IL-17 alone (*p* = 0.0061; Figure 4F) but no difference in IFN-γ positive subsets (Figure 4H).

## Discussion

To our knowledge, this is the first study in humans to determine differences in immunity in patients co-infected with TB and Influenza A/B compared to those with TB mono-infection at diagnosis. At presentation, 4.2% of our TB patients were co-infected with FluA/B. The TB/Flu co-infection rates are 80% higher than those reported in a recent study from South Africa showing an increased relative risk of death for TB/Flu co-infection compared to TB mono-infection (5) suggesting that Flu co-infection could be a major contributor to TB-related morbidity/mortality in our setting. We found an increase in both cellular and soluble cytokine profiles in co-infected subjects consistent with a pro-inflammatory response. Co-infected subjects also had increased bacterial load at the time of diagnosis suggesting a link between pro-inflammation and development of disease pathology. Future studies will aim to analyse this at the site of infection in TB patients and also to correlate with other clinical parameters such as chest x-ray score and spirometry.

We found a significant increase in MCP-1 (C-C Motif Chemokine Ligand 2 (CCL2), a key chemokine that regulates migration and infiltration of monocytes/macrophages to the site of infection) in TB/Flu co-infected compared to TB only patients following overnight stimulation of whole blood with PPD. Interestingly high CCL2 levels have also been linked with disease severity of TB patients (16) Innate immunity is a key signature in our cohort, with an increase in both phenotypic and functional (cytokine producing) CD4+ and CD8+ semi-invariant Vα7.2+CD161+ cells in TB/Flu co-infection. For CD8+ MAIT cells, this was mainly limited to IL-17 production, while total CD8+ T cells had significantly higher levels of IFN-γ+IL17- producing cells compared to TB mono-infected patients. Conversely, semi-invariant CD4+ cells (likely Germline-encoded mycosyl (GEM) cells) had significantly higher levels of IFN-γ+IL-17+ dual-producing cells and IFN-γ+IL-17- cells while total CD4+ cells had higher levels of dual-producing cells in co-infected patients compared to mono-infected. In a previous study of TB patients, accumulation of cells producing both Th1/Th17 cytokines correlated with disease severity (17) Together with the increase in bacterial load in co-infected patients, our findings suggest that concurrent infection with Influenza reduces anti-microbial immunity and increases disease severity in line with murine studies (12) Future studies should address changes following successful TB treatment to determine if anti-microbial immunity is restored and should also include a control group of non-TB subjects to determine the specificity of the response. Analysis of Type I interferon would also be important to assess the Type I/II balance in TB/Flu co-infection.

It was interesting to observe such a high rate of TB/Flu co-infection in our setting. Previous studies (7) have shown a Type I IFN signature that discriminates active TB from latent TB infection and this may therefore be reflecting some underlying co-infections. The major limitation of our study is the limited sample size of co-infected subjects and those with samples available for blood analysis. The cohort was not designed for TB/Flu as an end-point and subsequently we were limited in how many samples could be analyzed for immunological parameters. Future studies should be powered for TB/Flu as an endpoint now that incidence has been determined from this study.

In conclusion, we have clearly shown a modulation of immune response in patients co-infected with TB and Flu at presentation. Flu co-infection at TB diagnosis is associated with a higher bacterial load and differential cellular and soluble immune factors both in the lung (sputum) and blood. These findings show for the first time the impact of TB/Flu co-infection in a human cohort and suggest the benefit of Flu vaccination in TB-endemic settings.

## Author Contributions

JM, SJ, RH, and AB performed experiments and data analysis. BK contributed to manuscript preparation. JS designed experiments, analyzed data and wrote the manuscript.

### Conflict of Interest Statement

The authors declare that the research was conducted in the absence of any commercial or financial relationships that could be construed as a potential conflict of interest. The reviewer BS and the handling Editor declared their shared affiliation.
